# The Role of Exportin-5 in MicroRNA Biogenesis and Cancer

**DOI:** 10.1016/j.gpb.2017.09.004

**Published:** 2018-04-30

**Authors:** Ke Wu, Juan He, Wenchen Pu, Yong Peng

**Affiliations:** Department of Thoracic Surgery, State Key Laboratory of Biotherapy, West China Hospital, Sichuan University, Chengdu 610041, China

**Keywords:** Exportin-5, MicroRNA, Biogenesis, Dysregulation, Cancer

## Abstract

**MicroRNAs** (miRNAs) are conserved small non-coding RNAs that play an important role in the regulation of gene expression and participate in a variety of biological processes. The **biogenesis** of miRNAs is tightly controlled at multiple steps, such as transcription of miRNA genes, processing by Drosha and Dicer, and transportation of precursor miRNAs (pre-miRNAs) from the nucleus to the cytoplasm by **exportin-5** (XPO5). Given the critical role of nuclear export of pre-miRNAs in miRNA biogenesis, any alterations of XPO5, resulting from either genetic mutation, epigenetic change, abnormal expression level or posttranslational modification, could affect miRNA expression and thus have profound effects on tumorigenesis. Importantly, XPO5 phosphorylation by ERK kinase and its *cis*/*trans* isomerization by the prolyl isomerase Pin1 impair XPO5′s nucleo-to-cytoplasmic transport ability of pre-miRNAs, leading to downregulation of mature miRNAs in hepatocellular carcinoma. In this review, we focus on how XPO5 transports pre-miRNAs in the cells and summarize the **dysregulation** of XPO5 in human tumors.

## Introduction

**Figure 1:**
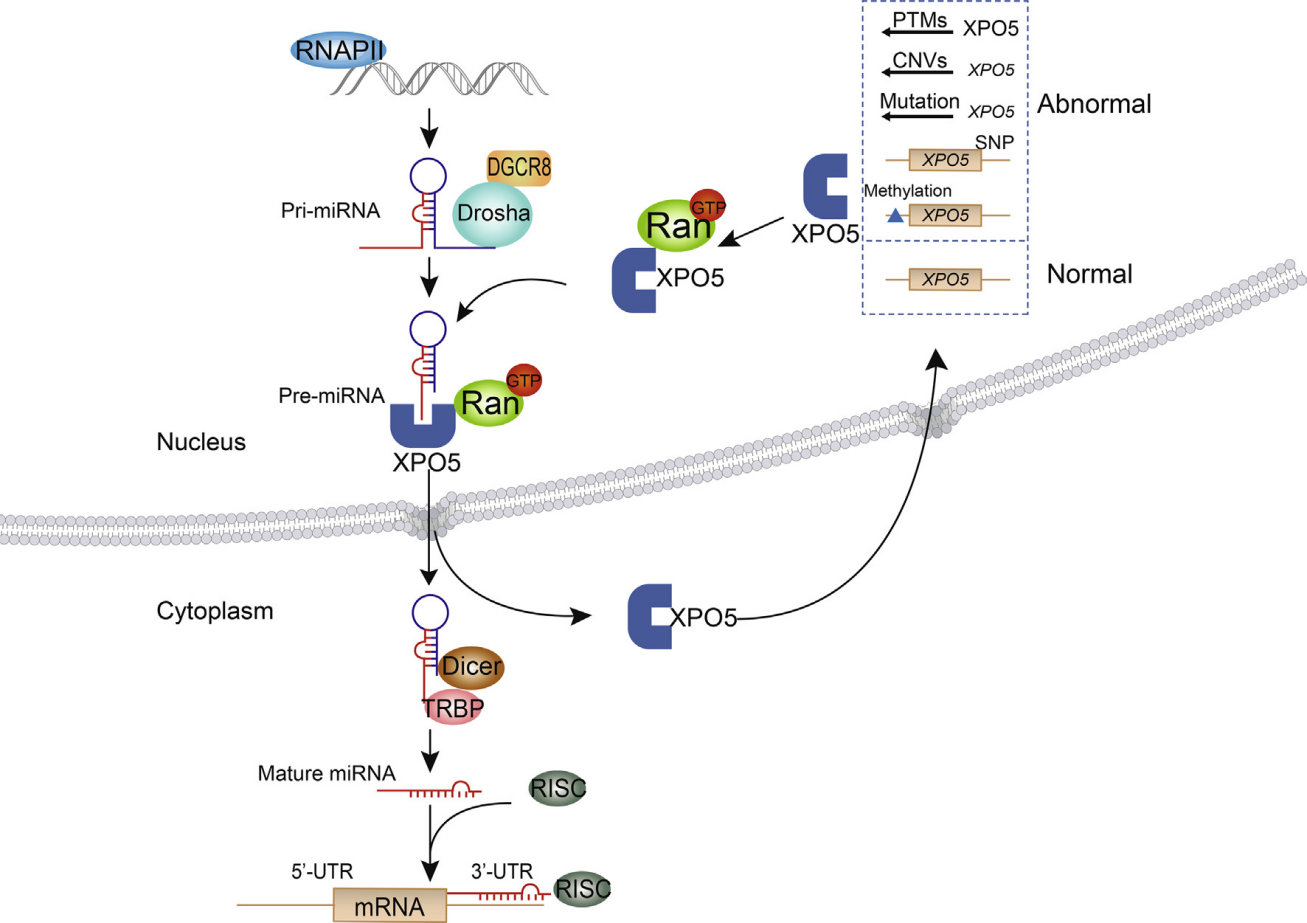
**The schematic diagram of miRNA biogenesis** miRNA genes are transcribed into pri-miRNAs by RNAPII. Then, the pri-miRNAs are cleaved by Drosha/DGCR8 complex into the pre-miRNAs. Following nuclear transport by Ran/GTP/XPO5 complex to the cytoplasm, the pre-miRNAs are processed by Dicer to produce mature miRNAs, which are ready to be incorporated into RISCs and regulate gene expression. Abnormality of *XPO5* or the encoded protein would affect the process of pre-miRNAs transport. RNAPII, RNA polymerase II; RISC, RNA induced silencing complex; XPO5, exportin-5; DGCR8, DiGeorge syndrome chromosomal region 8; PTM, post-translational modification; CNV, copy number variation; SNP, single nucleotide polymorphism.

MicroRNAs (miRNAs) are endogenous small non-coding RNAs, which are about 20–22 nucleotides (nt) in length and evolutionarily conserved among different species. Until now, 38,589 miRNA molecules in 271 species have been identified, including 1982 human precursor-miRNAs (pre-miRNAs) and 2694 human mature miRNAs (Release 22, http://www.mirbase.org). The miRNA biogenesis in mammals involves multiple stages (Figure 1). First, miRNA genes are transcribed, usually by RNA polymerase II (RNAPII), into primary transcripts (pri-miRNAs), which are 5′-capped and 3′-polyadenylated and contain imperfect hairpin structures. Then, the pri-miRNAs are cleaved by Drosha/DiGeorge syndrome chromosomal region 8 (DGCR8) complex into the pre-miRNAs with a ∼70-nt stem-loop structure. Following nuclear transport by GTP-binding nuclear protein Ran (RanGTP)/exportin-5 (XPO5) complex to the cytoplasm, the pre-miRNAs are processed by Dicer to produce the mature miRNAs. The mature miRNAs are then ready to be incorporated into the RNA induced silencing complex (RISC) and regulate gene expression through translational repression or mRNA degradation [1].

Although initially discovered in *Caenorhabditis elegans*, miRNAs have been a hot topic in biomedical field since Croce’s group for the first time revealed the frequent deletions and downregulation of *MIR-15* and *MIR-16* genes in human B cell chronic lymphocytic leukemia [2]. Moreover, human MIR genes are found to be frequently located at fragile sites and genomic regions that are involved in cancers. For example, MIR genes for miR-29a, miR-29b, miR-96, miR-182s, miR-182as, miR-183, and miR-129-1 were found in or close to the fragile site *FRA7H* [3]. High-throughput technologies have demonstrated that compared to normal tissues, there is a general downregulation of miRNA expression in multiple human tumors, which may be caused by defects in the miRNA biogenesis machinery [4], [5]. For instance, the expression of both Drosha and Dicer proteins, two key components of miRNA biogenesis, is significantly decreased in ovarian cancer specimens, and their expression levels are correlated with outcomes in patients with ovarian cancer [6]. Given that pre-miRNA transportation has been recently demonstrated to be critical for the generation of mature miRNAs, the impact of XPO5 on miRNA biogenesis and its role in tumorigenesis have attracted considerable attention in the field.

**Figure 2:**
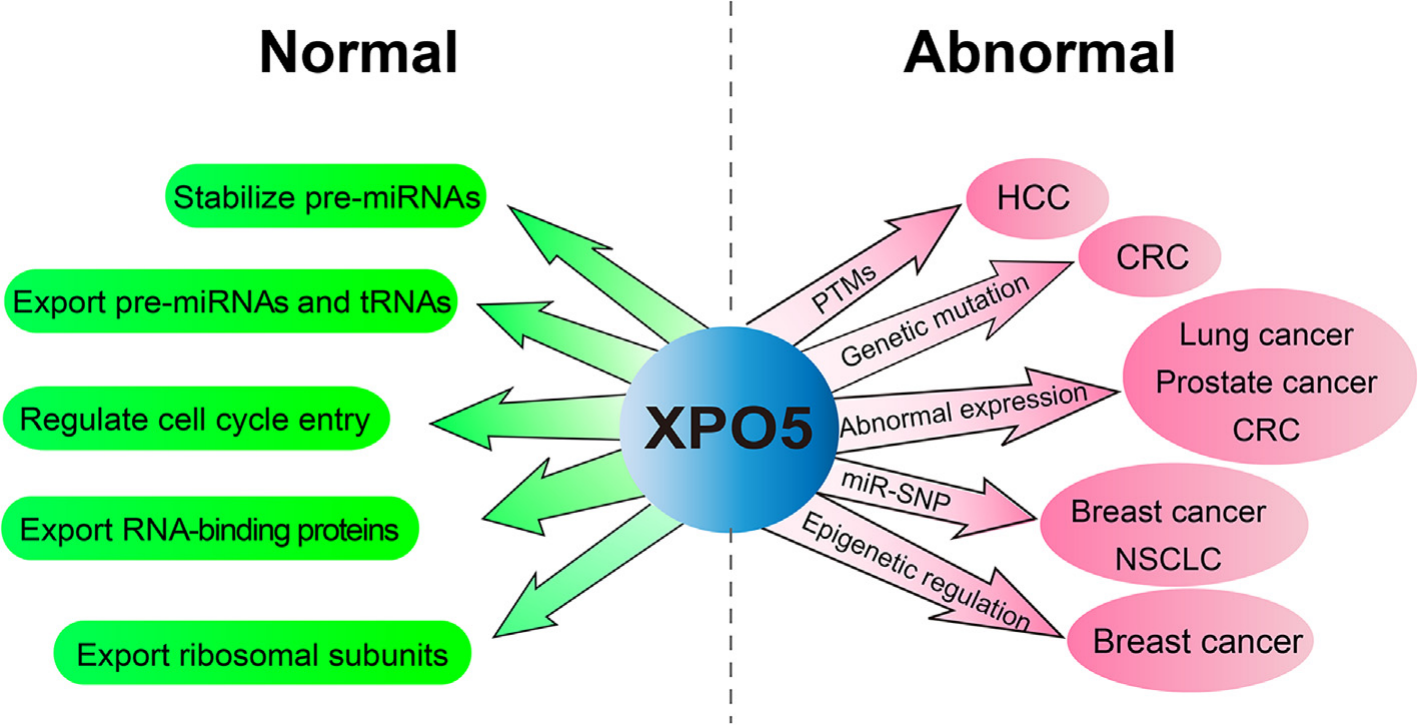
**Cellular function of XPO5** XPO5 participates in different cellular biological processes and is associated with various cancers. HCC, hepatocellular carcinoma; CRC, colorectal cancer; NSCLC, non-small cell lung cancer.

Increasing evidence indicates that XPO5 plays an important role in nuclear export of various substrates (Figure 2). During cell cycle entry, XPO5 is promptly induced to increase global miRNA expression through a PI3K-dependent post-transcriptional mechanism. Inhibition of XPO5 induction leads to a proliferation defect, which is associated with delayed G1/S transition [7]. Therefore, XPO5 acts as a critical molecular hub to control gene expression through regulating miRNA expression. Besides pre-miRNAs, XPO5 has been demonstrated to be involved in the export of some RNA-binding proteins (RBPs), such as Staufen homolog 2 (STAU2) and interleukin enhancer-binding factor 3 (ILF3), dependent on its double-stranded RNA-binding domain [8], [9], [10]. In addition, XPO5 is also shown to be required for nuclear export of 60S ribosomal subunit in a RanGTP-dependent process [11]. In this review, we briefly describe the role of XPO5 in miRNA biogenesis and discuss its dysregulation in cancer.

### Role of XPO5 in miRNA biogenesis

Exportin belongs to the karyopherin β family of transport factors, which mediate protein shuttling from the nucleus to the cytoplasm by recognizing specific transport signals in the cargo proteins. XPO5 is one of nucleo-cytoplasmic exportins and uses Ran-GTPase to control cargo association. XPO5 binds to pre-miRNAs at high Ran-GTP levels in the nucleus and transports the pre-miRNAs through the nuclear pore complex into the cytoplasm, where the pre-miRNAs are released from the Ran/GTP/XPO5 complex upon GTP hydrolysis. Subsequently, the free XPO5 in the cytoplasm returns to the nuclear compartment and mediates another round of pre-miRNA transport [12]. Since XPO5 is responsible for transporting pre-miRNAs from the nucleus to the cytoplasm, overexpression of XPO5 is found to improve transport efficiency and further enhance mature miRNA expression [13]. Therefore, XPO5-mediated nuclear export of pre-miRNAs could be a rate-limiting step during miRNA biogenesis.

**Figure 3:**
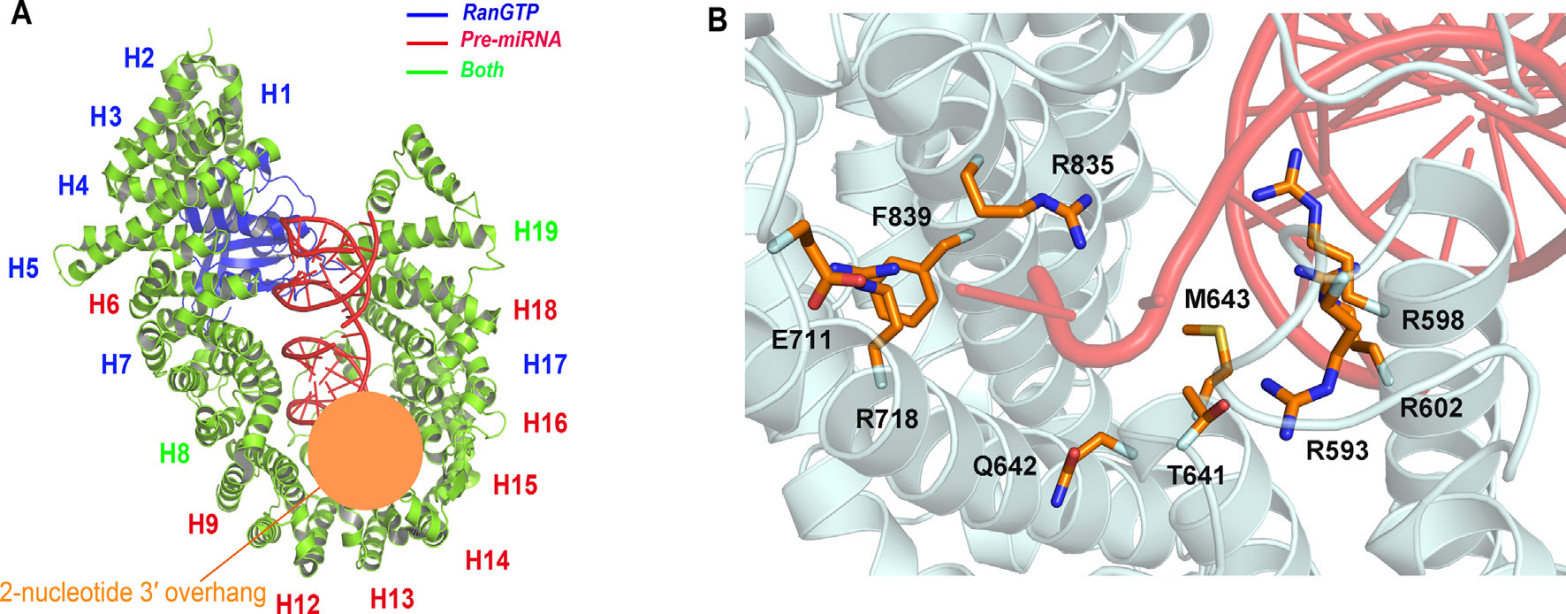
**The structure of the XPO5:RanGTP:pre-miRNA complex** **A.** Structure showing human pre-miRNA (red) bound to XPO5 (green) with RanGTP (blue). The stem moiety of a pre-miRNA is mostly covered by XPO5, and the 2-nt 3′overhang structure (highlighted in the orange circle) has many interactions with HEAT repeats 12-15 of XPO5. HEAT repeats 1-19 are indicated with a letter “H” and the corresponding numbers, which are shown in different colors based on their interacting partners (blue: interacting with RanGTP; red: interacting with pre-miRNA; green: interacting with both RanGTP and pre-miRNA). **B.** Intermolecular interaction details of the 2-nt 3′overhang structure of pre-miRNA(red) with HEAT repeats 12-15 of XPO5 (green). XPO5 PDB ID: 3A6P.

Using X-ray technology, Okada et al. has reported a 2.9 angstrom structure of the nuclear export machinery formed by pre-miRNA with complex of XPO5 and RanGTP [14]. XPO5 recognizes the double-stranded stem structure of the pre-miRNAs via the XPO5 tunnel-like structure comprising HEAT repeats (a tandem repeat protein structural motif composed of two alpha helices linked by a short loop) 8, 9, 12-16, 18, and 19. In detail, the tunnel-like structure of XPO5, shaped by R593, R598, R602, T641, Q642, M643, E711, R718, R835, and F839, interacts with 3′ overhang of a pre-miRNA via hydrogen bonds or salt bridges, protecting the pre-miRNA stem from hydrolysis by nucleases (Figure 3). Since the RNA binding by XPO5/RanGTP is not sequence-dependent, it is suggested that this nuclear export machinery can recognize a variety of pre-miRNAs [14].

Other than transporting pre-miRNAs from the nucleus to the cytoplasm, XPO5 also mediates nuclear export of eukaryotic elongation factor 1A (eEF1A) via aminoacylated tRNAs [15]. Intriguingly, XPO5 has been recently shown to regulate the expression of Dicer, an RNase III required for pre-miRNA maturation [16]. Mechanistically, XPO5 directly interacts with human *Dicer* mRNA, and knockdown of *XPO5* leads to an increased nuclear accumulation of *Dicer* mRNA in HeLa cells, thus decreasing Dicer protein expression [16]. Therefore, XPO5 plays an important role in miRNA biogenesis through both exporting pre-miRNAs and modulating Dicer expression.

## Dysregulation of XPO5 in cancers

Nuclear export of pre-miRNAs is accurately regulated in normal cells and its dysregulation could cause abnormal expression of mature miRNAs in cancer cells. Strikingly, more pre-miRNAs are found to be retained in the nucleus of both cancer cells and tumors, when compared with normal cells and normal tissues [17]. Using real-time PCR, Lee et al have profiled the expression of 225 pre-miRNAs and mature miRNAs in 22 different human tissues, 37 human cancer cell lines, as well as 16 pancreas and liver tissues/tumors. They find that a large number of MIR genes are transcribed and processed into pre-miRNAs, but not processed to mature miRNAs in cancer cells, indicating defects in the pre-miRNA nuclear export by XPO5 in human tumors [17]. The abnormal function of XPO5 could be caused by genetic or epigenetic change of *XPO5* as well as abnormal expression level or post-translational modifications (PTMs) of XPO5 protein (Table 1).

**Table 1:** The role of dysregulated XPO5 in different cancers

**Type**	**Description**	**Mechanism**	**Diseases**	**Refs.**
PTMs	Phosphorylation at T345/S416/S497 and *cis*/*trans* isomerization	Impair pre-miRNA export	HCC	[18], [19]

Genetic mutation	Generation of truncated XPO5 protein	Impair pre-miRNA export	CRC	[20]

Abnormal expression	Increase or decrease in XPO5 expression	Cause abnormal expression of mature miRNAs	Prostate adenocarcinoma, lung adenocarcinoma, CRC	[21], [22], [23], [24]

miR-SNP	Impact on individual’s response to certain drugs and risk of particular diseases	Unknown	Breast cancer, NSCLC	[25], [26], [27], [28]

Epigenetic regulation	Decrease in methylation of *XPO5* promoter region	Promote transcription	Breast cancer	[25]

*Note*: PTM, post-translational modification; SNP, single nucleotide polymorphism; HCC, hepatocellular carcinoma; CRC, colorectal cancer; NSCLC, non-small cell lung cancer.

### Abnormal expression of XPO5 protein

Abnormal expression of XPO5 protein has been found in human cancers. For example, compared to normal tissues, XPO5 expression is downregulated in bronchioloalveolar carcinoma and stage I adenocarcinoma [21], whereas it is about 1.6-fold upregulated in prostate adenocarcinoma [22], especially in the metastatic patients [23].

The alterations in XPO5 protein level lead to dysregulation of mature miRNA expression. Recently, XPO5 is found to act as an oncoprotein in colorectal cancer (CRC) due to its high expression in CRC and anti-tumor effect after *XPO5* knockdown [24]. Shigeyasu et al. reported an upregulated expression of XPO5 at both mRNA and protein levels in CRC samples when compared to normal tissues. Moreover, high XPO5 expression is associated with worse clinicopathological features and poor survival in CRC patients [24]. In SW480 and Caco-2 CRC cells, *XPO5* knockdown decreased the expression of key oncogenic miRNAs, such as miR-21, miR-10b, miR-27a, miR-92a, miR-182, and miR-155, resulting in decreased cell proliferation and invasion. In a xenograft animal model, silencing *XPO5* also significantly attenuates tumor growth [24]. Similarly, XPO5 expression is increased in breast tumors and urothelial carcinoma of the bladder [25], [29]. Therefore, XPO5 could act as an oncoprotein and may be a potential therapeutic target in certain cancers.

Interestingly, it is found that XPO5 expression can be regulated by miRNAs. For example, Li et al. report that overexpression of miR-138, a tumor suppressor miRNA, reduces XPO5 levels through downregulating the expression of required for meiotic nuclear division 5 homolog A (RMND5A), which is responsible for XPO5 stability [30]. Expression of miR-138 is often downregulated in tumors, such as head and neck squamous cell carcinoma, and nasopharyngeal carcinoma [31], [32], which may explain why XPO5 expression is upregulated in certain tumors.

### XPO5 protein PTMs

Protein PTMs, such as acetylation and phosphorylation, are considered as essential mechanisms to diversify the protein functions and dynamically coordinate their signaling networks in eukaryotic cells. Defects in PTMs of some proteins have been demonstrated in human diseases [33]. For the first time, Sun et al. discovered that XPO5 predominantly locates in the nucleus of hepatocellular carcinoma (HCC) cells, whereas in normal tissues it is distributed in both nucleus and cytoplasm [18]. When ERK kinase is activated in HCC cells, XPO5 is phosphorylated at T345/S416/S497 sites, which usually stay unphosphorylated in normal hepatocytes. Moreover, the prolyl isomerase Pin1 interacts with the phosphorylated XPO5 and mediates the change of its protein conformation through *cis*/*trans* isomerization, thus impairing the nuclear export of pre-miRNAs and leading to the retention of XPO5 in the nucleus [19]. As a consequence, such PTMs of XPO5 cause the decreased miRNA expression in HCC cells. These studies provide a novel mechanism to explain the downregulation of global miRNA expression in HCC. Interestingly, using the computer-aided drug design, Pu et al. identify a highly specific Pin1 inhibitor, API-1, and demonstrate that API-1 can effectively inhibit HCC growth both *in vitro* and *in vivo* [34]. Mechanistically, Pin1 inhibition by API-1 can upregulate miRNA biogenesis via retaining active XPO5 conformation, and suppress HCC development, indicating API-1 as a novel drug candidate for HCC therapy [34].

Besides phosphorylation, acetylation is identified at K396 site of XPO5 protein [35]. But the function of XPO5 acetylation still remains unknown.

### Genetic mutations of *XPO5* gene

Inactivating mutations in *XPO5* gene have been detected in a subset of human tumors with microsatellite instability, such as hereditary nonpolyposis colon cancer (HNPCC) [20]. In both HCT-15 and DLD-1 CRC cells, insertion of a single nucleotide “A” within *XPO5* gene generates a premature termination codon in exon 32 of *XPO5* mRNA. This frameshift mutation leads to the production of truncated proteins lacking the C-terminal region [20]. Because the C-terminal region of XPO5 is essential for the formation of pre-miRNA/XPO5/Ran-GTP ternary complex, the truncated XPO5 proteins can’t bind to and transport pre-miRNAs, thus leading to a nuclear accumulation of pre-miRNAs and a reduction in mature miRNA expression [20]. Interestingly, expression of wild-type *XPO5* in these cells reverses the impaired export of pre-miRNAs and inhibits cancer cell growth, suggesting that XPO5 may function as a tumor suppressor in CRCs [20]. As mentioned above, XPO5 could also function as an oncoprotein in CRCs [24]. Therefore, whether XPO5 function as a tumor suppressor or oncoprotein in CRCs is still debatable and needs further investigation.

### *XPO5* SNPs and CNVs

Single nucleotide polymorphism (SNP) is the most common type of genetic variations, which could be used to predict an individual’s response to certain drugs and risk of particular diseases [26]. Increasing evidence reveals the presence of SNPs in *XPO5* gene. For example, rs11544382 (M1115T) and rs34324334 (S241N), two missense SNPs, are reported in *XPO5* gene. The variant genotypes of rs11544382 are significantly associated with high breast cancer risk when compared to the common homozygous genotype [25]. Moreover, rs11077 (GRCh38.p7 chr6:43523209), a potential miRNA-associated SNP (miR-SNP) located in the 3′UTR of *XPO5* gene, has been extensively studied in various tumors. For example, rs11077 is associated with a better chemotherapeutic response in patients with metastatic colon cancer (AC genotype) [27]. The AC + CC genotypes of rs11077 are significantly associated with better survival and lower recurrence in NSCLC [28], whereas AA genotype of rs11077 is associated with better overall survival of SCLC patients [36]. Furthermore, multivariate analysis indicates that miR-SNP rs11077 of *XPO5* gene is an independent prognostic marker to predict survival for advanced SCLC and NSCLC patients [36], [37]. Other studies have also reported that patients with AA genotype of rs11077 exhibit an increased risk of esophageal cancer and gastric cancer [38], [39], and are associated with short overall survival in multiple myeloma (after autologous stem cell transplantation), Hodgkin’s lymphoma, and renal cell carcinoma [40], [41], [42]. The rs11077 associated miRNAs need further investigation.

Copy number variations (CNVs) are usually categorized into two main groups: short repeats and long repeats. Duan et al. comprehensively analyze SNPs and CNVs of the genes involved in miRNA processing, and find that there exist CNVs within the *XPO5* gene (Entrez ID:57510, Gene location(hg18):chr6:43598050-43651642(−), CNV ID:Variation_9530, CNV position(hg18):chr6:43583452-43604640, Observed CNVs:1 gain) [43]. In mammals, CNVs are the primary contributor to genome variability, thus modifying the diploid status of DNA [44]. To date, there is no direct evidence supporting that CNVs of *XPO5* are associated with any human diseases.

### Epigenetic regulation of *XPO5*

Epigenetic modifications including DNA methylation and histone modification are generally altered during tumorigenesis, which may trigger resistance to immune surveillance, chemotherapy, and targeted drugs [45], [46]. DNA hypermethylation at the CpG islands within gene promoter usually leads to gene silencing. Leaderer et al. have reported the decreased methylation in the CpG island spanning the promoter region, the first exon, and part of the first intron of the *XPO5* gene (−600 to 808), which may explain why XPO5 expression is upregulated in breast cancer [25]. Whether RNA modification or histone acetylation could affect *XPO5* gene expression still remains elusive.

## Concluding remarks

Nuclear export of pre-miRNA by XPO5 is a critical step for miRNA biogenesis, so XPO5 expression requires accurate regulation to maintain its normal function under physiological conditions. Recently, genetic or epigenetic change of *XPO5* gene as well as abnormal expression level or PTMs of XPO5 protein, potentially leading to abnormal expression of mature miRNAs, have been found to be associated with certain tumors. However, the underlying mechanisms still remain elusive and need further investigation. For example, XPO5 expression is upregulated in CRCs and prostate cancer, but downregulated in lung adenocarcinoma. It seems that XPO5 may exert its function depending on tissue-specific expression of miRNAs. Such issues could be addressed by deep sequencing and proteomics technologies. Moreover, whether XPO5 functions as a tumor suppressor or oncoprotein in certain cancers should be investigated in animal model. Based on the findings that XPO5 can transport certain tRNAs and proteins besides pre-miRNAs, discovery of other XPO5 substrates could be helpful to understand the role of XPO5 in different tumors. Although PTMs of XPO5 such as acetylation [35] have been identified, their functional implications still remain unclear. Therefore, further investigations are needed to dissect the role of XPO5 during tumorigenesis and its underlying molecular mechanisms.

## Competing interests

The authors declare that they have no conflicts of interest with the contents of this article.
